# Nonoperative Management of an Acute Complete Achilles Tendon Rupture With Mesenchymal Stem Cells and Extracellular Vesicles: A Case Report

**DOI:** 10.7759/cureus.118122

**Published:** 2026-10-18

**Authors:** Abdiel Ortiz, Christian Diaz

**Affiliations:** 1 Orthobiologics, Auragens, Panama City, PAN; 2 Advanced Cellular Therapies, Auragens, Panama City, PAN

**Keywords:** achilles tendon rupture, allogenic, case report, exosomes, extracellular vesicles, mesenchymal stem/stromal cells, nonoperative management, orthobiologics, regenerative medicine, umbilical cord

## Abstract

An acute complete rupture of the Achilles tendon with a large intertendinous gap is conventionally regarded as a surgical indication, yet operative repair carries a significant burden on wound-healing and potential for neurovascular complications, whereas nonoperative care may be limited by re-rupture and incomplete functional recovery. Human umbilical cord-derived mesenchymal stem/stromal cells (hUC-MSCs) and their secreted extracellular vesicles (EVs) exert immunomodulatory, pro-angiogenic and pro-tenogenic paracrine effects that could support tendon healing.

A healthy 45-year-old recreationally active male sustained an acute complete rupture of the right Achilles tendon while playing tennis. A 3.0-T magnetic resonance imaging (MRI) confirmed a full-thickness mid-substance rupture located 6.1 cm proximal to the calcaneal insertion, with a 3.7 cm gap. He delayed treatment and opted out of surgical management and was offered nonoperative care with a combined biologic regimen: two ultrasound-guided intralesional injections of hUC-MSC-derived EVs (a total of 30 × 10⁹ vesicles) deposited into the tendon gap, both stumps, and the peritendon along with three consecutive daily intravenous (IV) infusions of hUC-MSCs (approximately 1.7 × 10⁶ cells/kg of total body weight) as a paracrine priming adjuvant, alongside an injury-guided functional rehabilitation program.

Pain measured using the numeric rating scale (NRS) decreased from 10/10 at baseline to 0/10 at six months post-intervention. At final follow-up (approx 14 months) the patient had returned to regular weight training and jogging without pain or functional limitations. On physical examination, the Thompson Test was negative, the Achilles Tendon Total Rupture Score (ATRS) and the American Orthopaedic Foot & Ankle Society (AOFAS) ankle-hindfoot scores were both calculated as 100/100, and instrumented biomechanical testing showed near-symmetric lower-limb range of motion and balance. Follow-up MRI demonstrated a remodeled tendon with restoration of continuity and no residual gap. No mild or severe adverse events were reported during or after procedures.

In this single case report, a combined regimen of systemic hUC-MSCs and intralesional hUC-MSC-derived EVs was associated with imaging-confirmed tendon healing and full functional recovery of an acute complete Achilles tendon rupture managed nonoperatively. This treatment protocol serves as a hypothesis-generating observation with potential for validation in controlled prospective studies.

## Introduction

Acute Achilles tendon rupture (ATR) is among the most common tendon injuries, with a rising incidence that disproportionately affects recreationally active adults in their third to fifth decades of life [1,2]. The optimal management for ATR remains debated; contemporary randomized and meta-analytic evidence indicates that, when combined with early functional rehabilitation, nonoperative management yields functional outcomes comparable to surgery while avoiding operative complications, albeit with the risk of a modestly higher re-rupture rate [3-5]. A network meta-analysis of 2,060 patients confirmed that the balance of re-rupture versus wound and neurovascular complications is highly sensitive to the specific combination of treatment and rehabilitation employed [1].

Most of this evidence, however, derives from ruptures amenable to apposition of the proximal and distal tendon ends. A complete rupture with a large intertendinous gap and stump retraction, as presented in this case subject, is generally considered a surgical indication, because a persistent gap is thought to compromise the mechanical continuity required for high-quality healing [2]. The Achilles tendon is nonetheless a hypovascular, bradytrophic tissue whose intrinsic healing frequently proceeds through disorganized scar rather than true regeneration, predisposing to elongation, stiffness, and re-injury regardless of the treatment alternative [6,7].

These biological limitations have motivated interest in orthobiologic augmentation of injury repair by exploiting autologous regenerative strategies such as platelet-rich plasma (PRP) and bone marrow-derived mesenchymal stem/stromal cells (MSCs) [2]. In this case we chose to employ an allogeneic source of MSCs which mainly act as medicinal/paracrine signaling cells rather than by engraftment, secreting trophic, immunomodulatory, and pro-angiogenic mediators and migrating toward injured tissue to orchestrate repair via chemotaxis and fibroblast mobilization [8,9]. A substantial part of this paracrine activity is conveyed by MSC extracellular vesicles (EVs), including the exosome subpopulation, which are payloads of proteins, lipids, and regulatory mRNA transferred to neighboring cells [10]. In preclinical tendon-injury models, MSC and tendon stem cell-derived EVs have been shown to attenuate the early inflammatory response, shift macrophage polarization toward a pro-regenerative phenotype (M2 phenotype), enhance tenocyte proliferation and migration, and improve collagen organization and biomechanical strength [10-15]. Human umbilical cord-derived MSC (hUC-MSC) exosomes have also demonstrated the ability to direct tendon regeneration through mTOR signaling, and purified exosome products improve intrinsic tendon regeneration in vivo [16,17]. Systematic reviews of in vivo and preclinical studies consistently support the pro-healing effect of MSC-EVs in tendon and specifically in Achilles tendon repair, lending support to the rationale for using these biologic products in this particular case [11,13,14].

Despite this mechanistic rationale, clinical translation for complete ATR is limited, and to date reports have yet to describe in vivo application of an allogeneic mesenchymal stem cell source for this particular use case. We chose to use umbilical cord-derived MSCs and their EVs due to our access to the biologic product through the manufacturing institution. The therapeutic profile of these cells in vitro is comparable to other cell sources; furthermore, their derived exosomes also favor therapeutic benefits in tendon and bone healing [14,16]. Furthermore, umbilical cord-derived cells exhibit a distinct secretome profile that is inherently teno-protective, promoting the modulation of inflammation via macrophage polarization towards an anti-inflammatory M2 phenotype [18]. The treatment protocol included ultrasound-guided intralesional delivery of hUC-MSC-derived EVs directly into the rupture site as well as intravenous (IV) application of hUC-MSC intended to systemically modulate the inflammatory response as an adjuvant to the local healing process, thereby promoting a favorable microenvironment for tissue remodeling. We report the imaging and functional outcomes of such a combined, fully nonoperative regimen in an acute complete Achilles rupture with a large gap. This report abides by the CAse REport (CARE) guidelines and describes the feasibility, safety, imaging findings, and functional outcomes following this combined nonoperative approach.

## Case presentation

Patient information

A 45-year-old male (BMI 24.4 kg/m²), an office-based business professional who exercised recreationally (gym training, tennis and occasional running), presented after an acute injury to the right lower limb while playing tennis non-competitively. His only relevant medical history was adolescent epilepsy managed with levetiracetam (750 mg twice daily from 2006 to 2009), subsequently discharged and seizure-free without medication. Patient has no history of drug, alcohol or tobacco consumption, and no history of prior tendinopathy, fluoroquinolone or systemic corticosteroid exposure, trauma or localized injections in the right ankle/foot region. 

Clinical findings and medical history

On 17 November 2024, while running during a recreational tennis match, the patient experienced a sudden audible “snap” originating from his right heel accompanied by severe pain and immediate inability to bear weight. He limited initial management to physical measures (cold compresses), over-the-counter nonsteroidal anti-inflammatory drugs (NSAIDs), and delayed medical evaluation to three weeks after the injury occurred.

Orthopedic examination on presentation revealed posterior leg and heel swelling with ecchymosis and a palpable tendon defect; Thompson and Matles tests were both positive with an inability to perform single-limb heel rise.

Diagnostic assessment

Magnetic resonance imaging (MRI) of the right ankle, shown in Figure 1, performed on a 3.0-T system (multiplanar T1-, T2- and proton-density-weighted sequences with fat saturation) in a set-neutral position demonstrated a complete rupture of the Achilles tendon located approximately 6.1 cm proximal to its calcaneal insertion, with a 3.7 cm tendinous gap filled by inflammatory fluid, as reported by an independent, third-party radiologist. Ancillary findings included myotendinous junction, myofibrillar edema with peri-soleal fluid, interstitial subcutaneous edema of the posterior compartment and plantar foot, fluid surrounding the sural nerve, Grade I sprains of the anterior and posterior talofibular and deltoid ligaments, and tenosynovitis of the posterior tibialis, flexor digitorum longus, and flexor hallucis longus tendons. The findings were consistent with an acute complete mid-substance ATR with a large gap, a lesion typically derived to operative repair [5]. 

**Figure 1:**
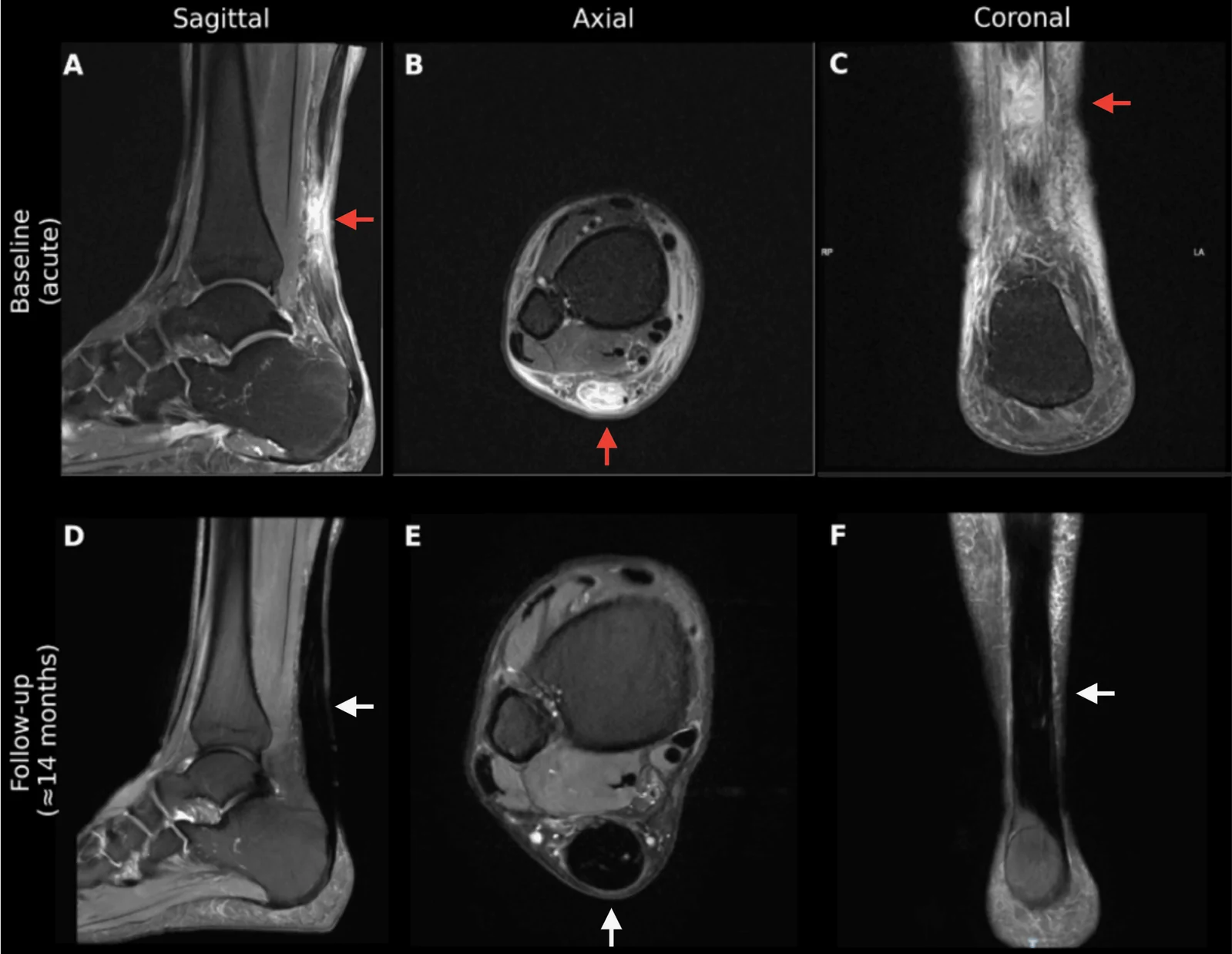
Baseline and follow-up 3.0-T MRI of the right ankle. (A-C) Baseline study obtained in the acute phase: fat-suppressed sagittal (A), axial (B), and coronal (C) images demonstrate a full-thickness rupture of the Achilles tendon approximately 6.1 cm proximal to the calcaneal insertion, with a 3.7 cm gap occupied by high-signal inflammatory fluid, myotendinous junction edema, and posterior soft-tissue edema. Red arrows show the tendon rupture. (D-F) Follow-up study at approximately 14 months following intervention: sagittal (D), axial (E), and coronal (F) images demonstrate a continuous, fusiform thickened tendon with residual intrasubstance signal consistent with remodeling scar, without residual gap, fluid collection, or evidence of re-rupture. White arrows show the absence of a prior tendon rupture defect. Images were cropped and scaled for display; corresponding views are not presented at identical magnification.

Biologic products and biomanufacturing

Allogeneic products (hUC-MSCs and EVs) were biomanufactured under standardized conditions by the treating institution in an American Association for Blood and Biotherapies (AABB)-accredited laboratory. Isolation of MSCs from human umbilical cord tissue (Wharton’s Jelly) were donated under informed consent of a single donor mother and subjected to rigorous testing to ensure sterility, characterization, viability and potency as defined by International Society of Cell and Gene Therapy (ISCT) MSC criteria [19]. MSCs were characterized using flow cytometry to confirm surface expression markers (CD90+, CD105+, CD73+, and CD44+) and the absence of hematopoietic markers (CD34-, CD11b-, CD45-, CD19-) and HLA-DR-. Quality control criteria were measured via viability, quantification, sterility, and endotoxin testing during cryopreservation, thawing, and syringe preparation as listed in Table 1. MSC-derived EVs were isolated and purified from culture media using ultracentrifugation techniques and quantified using electron microscopy and size exclusion criteria of 80 to 130nm in size.

**Table 1:** Characteristics of human umbilical cord mesenchymal stem cells. hUC-MSC: human umbilical cord-derived mesenchymal stem cells, HTLV: human T-cell lymphotropic virus, HIV: human immunodeficiency virus, VDRL: venereal disease research laboratory, ID+AB: identification and antibiotic susceptibility, CD: cluster of differentiation, MSC: mesenchymal stem cells

Parameter	Result
Product identity	
Cell type	hUC-MSC
Lot code	AUC07FEB24 C2
Dose per vial (n=15)	10 × 10⁶ cells
Passage # at harvest	≤ 5
Release criteria	
Cell viability	Pass
Endotoxin	Pass
Donor infectious disease screening	
Cytomegalovirus	Non-reactive
Hepatitis B	Non-reactive
Hepatitis C	Non-reactive
HTLV I and II	Non-reactive
Toxoplasma gondii	Non-reactive
HIV 1+2	Non-reactive
VDRL	Non-reactive
Microbiological testing	
Aerobic and anaerobic culture (ID+AB), 96 h	Negative
Mycoplasma	Negative
Fungus	Negative
Immunophenotype	
CD90⁺	92.80%
CD73⁺	95.50%
CD105⁺	94.30%
CD44⁺	93.20%
Non-MSC phenotype	0.80%

Ultrasound-guided intralesional EV therapy

Local therapy comprised two ultrasound-guided intralesional injections of hUC-MSC-derived EVs on December 12 and 20, 2024, respectively (15 × 10⁹ vesicles per injection preparation; a total of 30 × 10⁹ vesicles). For each session the patient was placed in the prone position; keeping sterility, the skin was prepared with 4% chlorhexidine-isopropyl alcohol (ChloraPrep; Becton, Dickinson and Company, Franklin Lakes, NJ, USA) and local subcutaneous (SC) lidocaine 2% as an anesthetic agent. With the use of the ultrasound’s linear transducer along the long-axis, in-plane approach, one (15 × 10⁹ vesicles) vial of EVs suspended in 8 mL of Lactated Ringer’s solution was injected under real-time visualization into the right Achilles tendon’s proximal and distal stumps, the peritendon sheath and the intralesional gap. Real-time ultrasound images taken during procedures can be seen in Figure 2 and illustrated in Figure 3 for better comprehension. 

**Figure 2:**
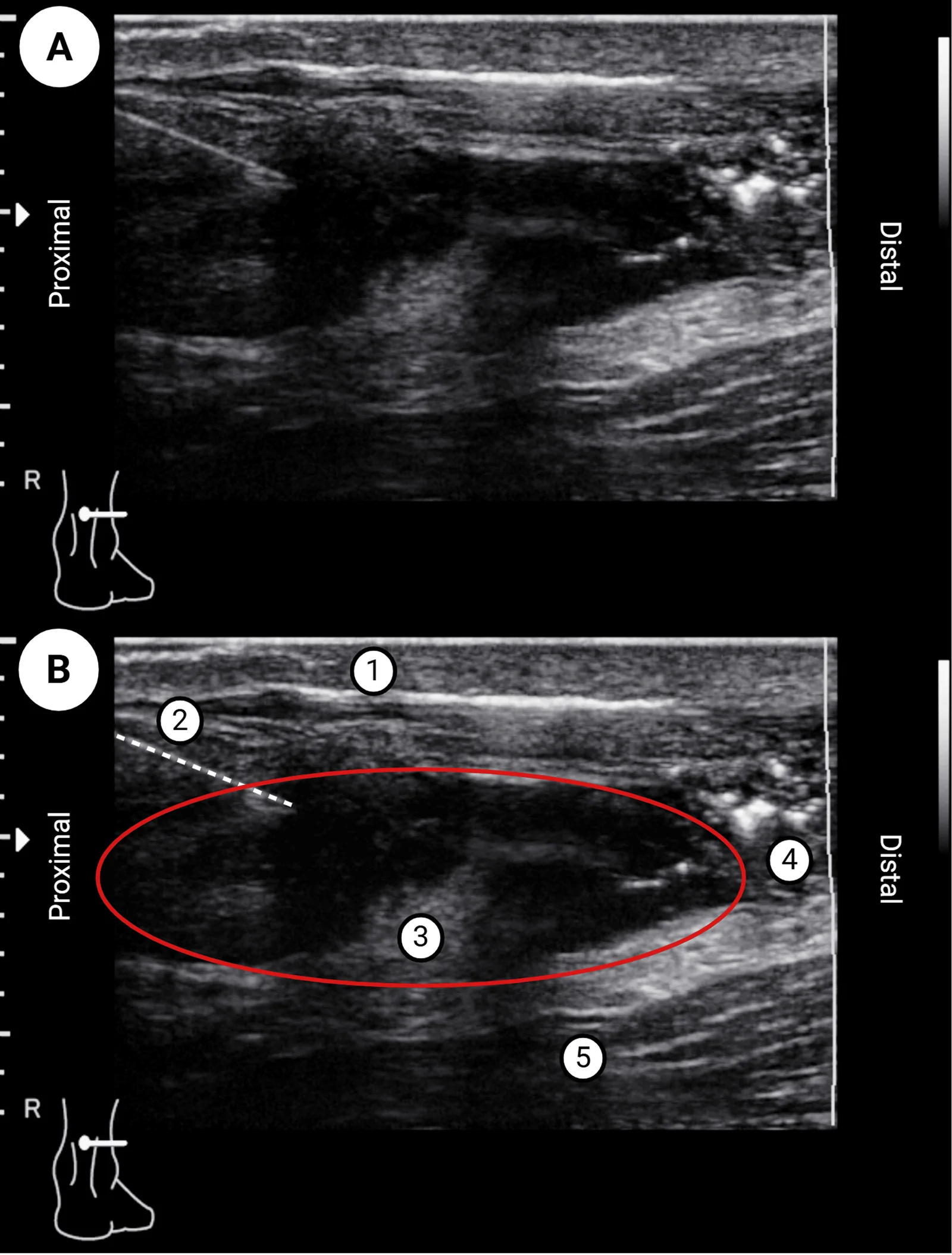
Ultrasound-guided, long-axis (in-plane) intralesional injection of hUC-MSC-derived EVs. (A) unlabeled B-mode reference image and (B) annotated image: (1) skin/paratenon (superficial margin); (2) needle in long-axis, in-plane trajectory; (3) complete rupture gap/defect filled by hypoechoic hematoma/granulation tissue; (4) already-deposited EVs seen as hyperechoic injectate; (5) Kager’s fat pad and deep posterior compartment. hUC-MSC: human umbilical cord-derived mesenchymal stem cells, EVs: extracellular vesicles

**Figure 3:**
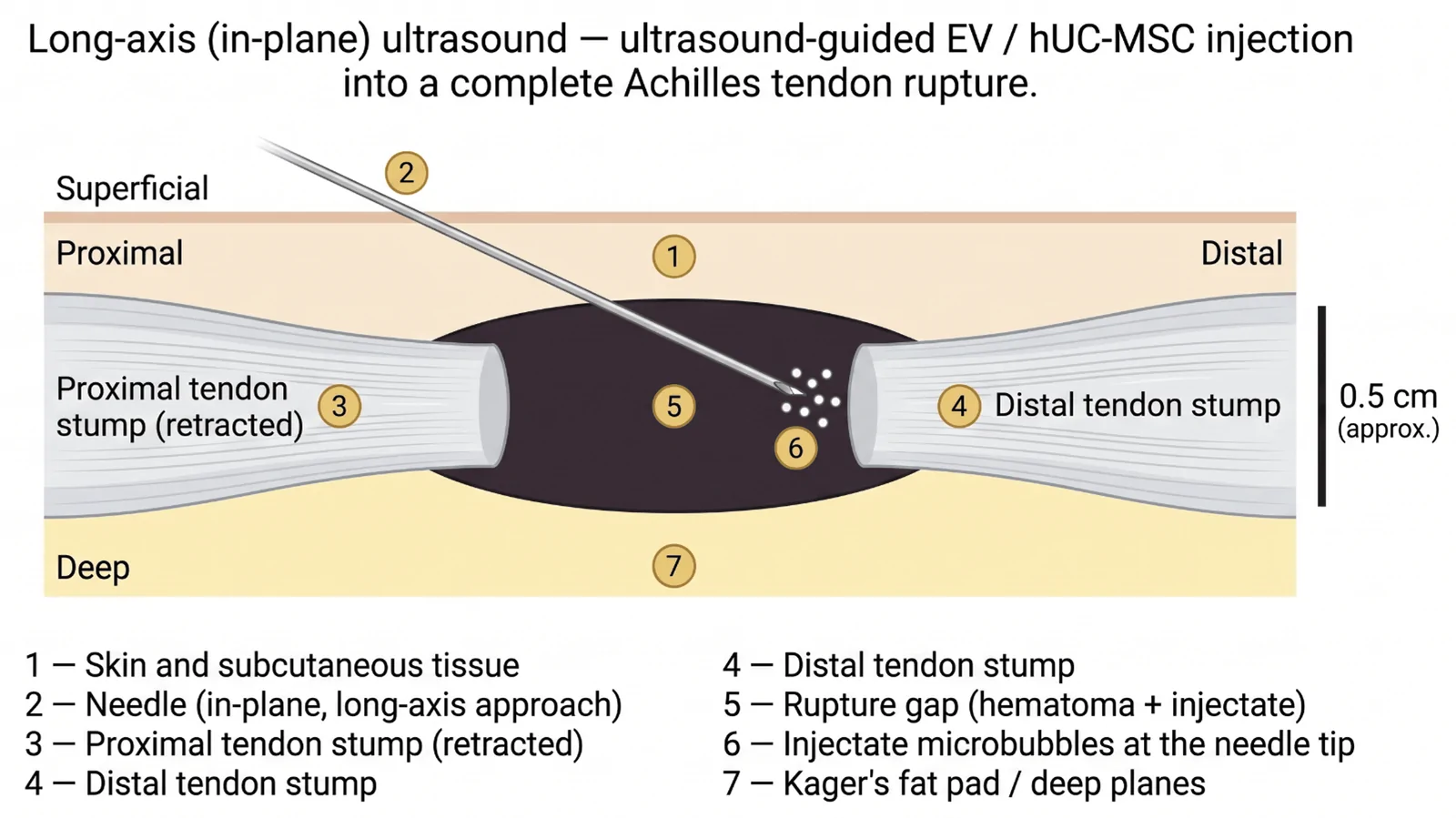
Schematic of the injection distributing EVs into the proximal stump, distal stump, peritendon and intralesional gap. Created in BioRender. Diaz, C. (2026) https://BioRender.com/bjaqxuv hUC-MSC: human umbilical cord-derived mesenchymal stem cells, EVs: extracellular vesicles

Systemic hUC-MSC therapy

The patient received systemic administration of hUC-MSCs as an adjunct to the localized EV therapy, with the rationale of modulating the inflammatory response systemically while supporting the local reparative microenvironment. This combined strategy was intended to promote coordinated tissue remodeling and enhance tendon healing. The protocol consisted of three consecutive daily IV infusions of hUC-MSCs on December 2, 3, and 4, 2024; 50 × 10⁶ cells per dose, a total of 150 × 10⁶ cells; the patient weighed 88.5kg (195lbs) and measured 190.5cm (6'3'') yielding a BMI of 24.4kg/m^2^; a nominal concentration 1.7 × 10⁶ cells/kg total body weight (TBW). Infusions were administered via peripheral vein cannulation in a clinical setting as supervised by nursing and medical staff, and were well tolerated without infusion-related reactions or reported adverse events. 

Rehabilitation

A four-phase functional rehabilitation program accompanied biologic therapy. Phase 1 (weeks 0-2) involved protected immobilization in a walker boot with a 20° plantarflexion heel wedge. Phase 2 (weeks 2-6) consisted of continued protected immobilization (boot with 20° wedge) plus magnetotherapy, therapeutic ultrasound, and neuromuscular electrostimulation to promote local capillarity and trophism. In Phase 3 (month 2-3), the patient transitioned the boot to a neutral position starting at week 8, initiating progressive partial weight-bearing (50% of total body weight with crutches), continuing the physical modalities. Finally, Phase 4 (month 3-12) incorporated progressive passive and active-assisted range-of-motion work, resistance exercise, proprioceptive and balance training, progressive strengthening from isometric to isokinetic loading, gait re-education, and plyometrics, with adjunctive magnetotherapy, therapeutic ultrasound and electrostimulation. Complete timeline of events can be seen in Table 2.

**Table 2:** Chronology of the injury, combined biologic intervention, rehabilitation and outcome assessments (CARE timeline) hUC-MSC: human umbilical cord–derived mesenchymal stem/stromal cell, EV: extracellular vesicle, MRI: magnetic resonance imaging, AOFAS: American Orthopaedic Foot & Ankle Society, ATRS: Achilles Tendon Total Rupture Score.

Date / timepoint	Event
17 Nov 2024	Acute injury: audible snap in right heel while playing tennis; severe pain, inability to bear weight, posterior leg/heel swelling and ecchymosis.
Early Dec 2024 (≈3 weeks post-injury)	Clinical evaluation: palpable defect, positive Thompson test; nonoperative biologic protocol elected after informed consent.
2-4 Dec 2024	Systemic therapy: three daily intravenous hUC-MSC infusions (50 × 10⁶ cells/day; total 150 × 10⁶).
6 Dec 2024	Baseline 3.0-T MRI: complete mid-substance rupture 6.1 cm from insertion, 3.7 cm gap (Figure 1).
12 Dec 2024	First ultrasound-guided intralesional EV injection (15 × 10⁹ vesicles) into gap, stumps and peritendon (Figure 2).
20 Dec 2024	Second ultrasound-guided intralesional EV injection (15 × 10⁹ vesicles).
Weeks 0-12	Phased protected rehabilitation: boot with 20° wedge → neutral with progressive weight-bearing; physical modalities.
9 months	Negative Thompson test; Matles test preserved; full load tolerance (hop/run/jog).
4 Feb 2026 (≈14 months)	Follow-up MRI: restored tendon continuity, no residual gap (Figure 1).
20-21 Feb 2026	AOFAS/ATRS 100/100; symmetric instrumented biomechanics (VALD); full return to activity.

Follow-up and outcomes

Pain intensity, rated on a 0-10 numeric rating scale (NRS), fell from 10/10 at baseline evaluation to 6/10 at two weeks post-intervention, to 4/10 at three months, and 0/10 at both six months and 12 months evaluations, graphed for illustrative purposes in Figure 4. Given that early immobilization is a traditional approach for managing complete Achilles rupture, ankle range of motion and plantarflexion strength were deliberately not tested during the early phases due to pain and swelling; these were assessed only after the tendon had healed.

**Figure 4:**
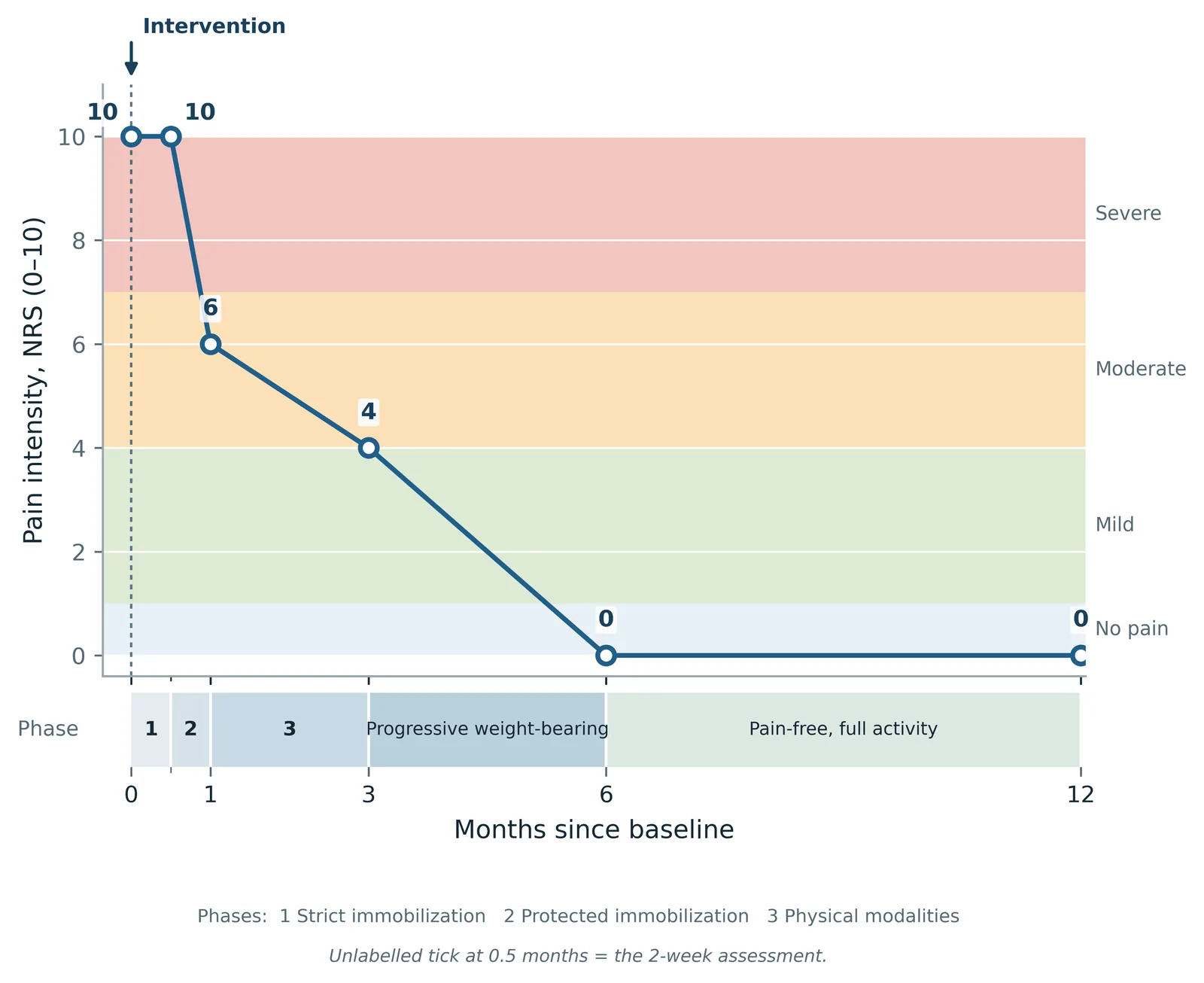
Pain trajectory on a 0-10 numeric rating scale (NRS) across follow-up time points. *Range of motion and strength were not assessed at early timepoints owing to protective immobilization. Created by the authors using Python (Matplotlib), with AI-assisted coding (Claude; Anthropic, San Francisco, CA, USA). All values were verified against the clinical record.

At nine months the Thompson (Simmonds-Thompson) calf-squeeze test was negative and the Matles test demonstrated preserved resting tension with active plantarflexion, both consistent with tendon continuity. Load testing (hopping, running, and jogging) demonstrated full capacity. Formal outcome assessment at approximately 12-14 months yielded both Achilles Tendon Total Rupture Score (ATRS) [20] and American Orthopaedic Foot and Ankle Society (AOFAS) [21] ankle-hindfoot scores of 100/100 (AOFAS: pain 40/40, function 50/50, alignment 10/10). Instrumented three-dimensional biomechanical assessment (VALD; VALD Performance, Brisbane, Australia) performed on 20 February 2026 showed near-symmetric lower-limb kinematics: peak ankle dorsiflexion during a bilateral squat was 39° on the treated (right) side versus 43° on the left (9% asymmetry), single-leg balance (95% center-of-mass ellipse area) was symmetric (right 369 mm² vs left 348 mm²), and single-leg squat knee flexion was symmetric (right 55° vs left 58°); the only residual finding was a modest dorsiflexion deficit of the right ankle during a deep forward lunge (21° vs 28°), an asymmetry of 25%. VALD biomechanical assessment measurements comparing right versus left lower limbs can be seen in Table 3. Clinical examination confirmed symmetric active range of motion between limbs can best be evaluated in Figure 5. 

**Table 3:** Instrumented three-dimensional biomechanical assessment (VALD) at approximately 14 months. The right limb is the injured (treated) side. Asymmetry is expressed relative to the higher of the two limb values. For the single-leg-stand balance task a larger 95% centre-of-mass (CoM) ellipse area indicates greater postural sway.

Task / metric	Left	Right (affected)	Asymmetry
Squat - peak ankle dorsiflexion (°)	43	39	9%
Forward lunge - peak ankle dorsiflexion (°)	28	21	25%
Single-leg squat - peak knee flexion (°)	58	55	5%
Single-leg stand - 95% CoM ellipse area (mm²)	348	369	6%

**Figure 5:**
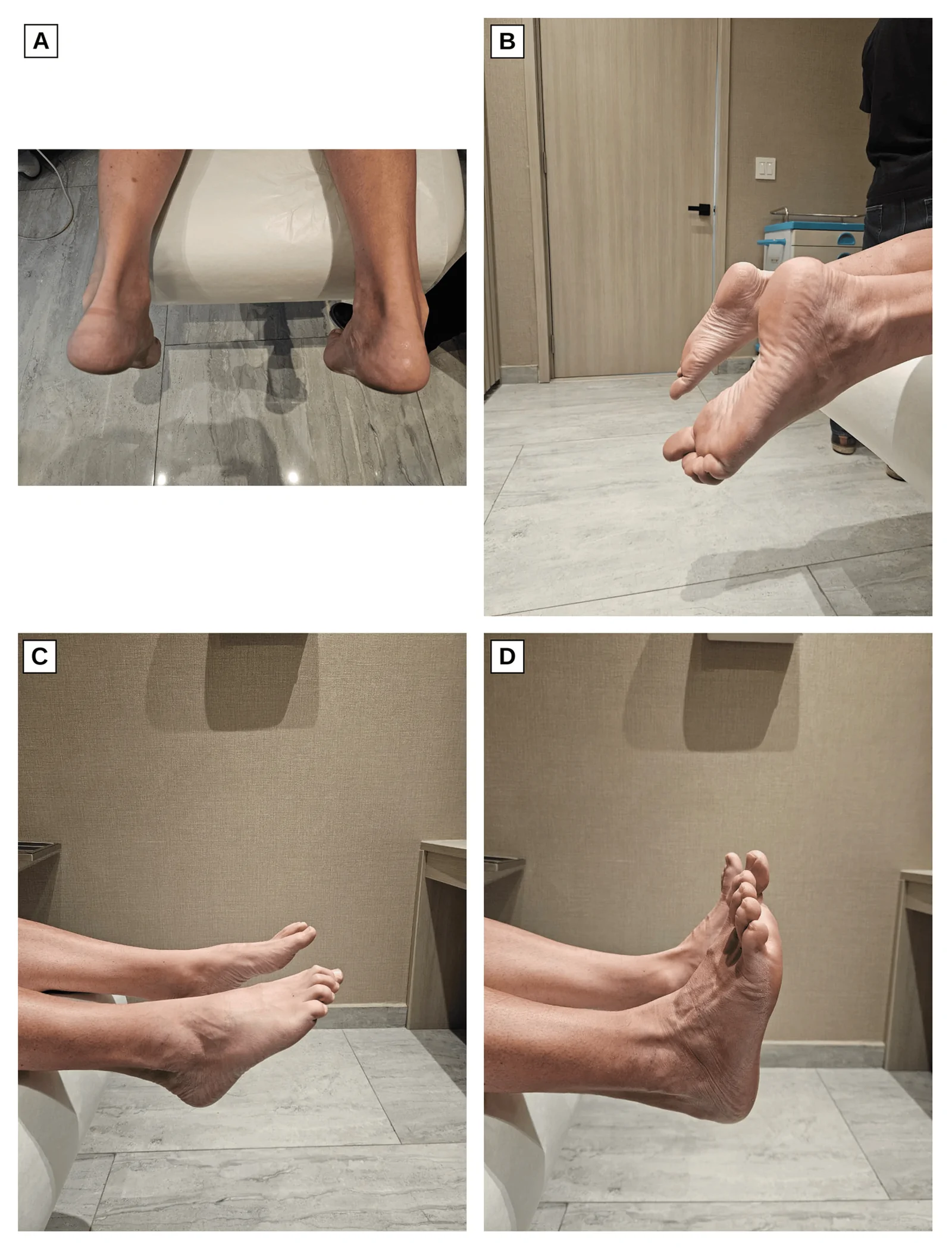
Clinical functional recovery at final follow-up. (A) Bilateral view of both ankles showing a symmetric, preserved hindfoot and Achilles-region contour without deformity; (B) symmetric bilateral active plantarflexion; (C) lateral view of active plantarflexion; (D) full active and symmetric dorsiflexion of the treated limb. Symmetric heel-rise and plantarflexion capacity are consistent with a healed, functionally competent tendon.

Follow-up MRI of the right ankle, as seen in panels D, E, F of Figure 1, and marked by a white arrow, demonstrated restoration of tendon continuity: the Achilles tendon was thickened with discrete intrasubstance signal change in its mid-distal portion, without an identifiable separation of fibers, residual gap or fluid collection - findings interpreted as a healed, remodeling tendon (intrasubstance/tendinopathic signal) rather than a persistent tear. Incidental findings included mild inflammatory changes of the anterior tibiofibular and talofibular ligaments and a prominent posterior trigonal process with marginal osteophytes. No re-rupture was observed. 

At final follow-up (Figure 5) the patient had returned fully to his prior activities (gym training and jogging) with no residual limitation, rated his satisfaction as excellent, and experienced no complications throughout the treatment and rehabilitation course.

## Discussion

This case documents the functional healing of an acute complete Achilles rupture with a large (3.7 cm) gap, confirmed by gold-standard imaging studies and clinical follow-up. This lesion is indicated for surgical repair, hence, opting for an entirely nonoperative regimen that combined systemic hUC-MSC infusion with ultrasound-guided intralesional hUC-MSC-derived EVs and structured rehabilitation shows feasibility in this case. Restoration of tendon continuity on MRI, a negative Thompson test, AOFAS/ATRS scores of 100/100, and near-symmetric instrumented biomechanics together indicate a favorable structural and functional result without the wound-healing, sural-nerve injury, and potential complications associated with operative repair [1,2].

The biological rationale for this combined approach is grounded in the paracrine activity paradigm underlying MSC therapy. Rather than engrafting and differentiating, MSCs home to injured tissue and function as medicinal signaling cells that secrete an immunomodulatory and pro-angiogenic secretome packaged within their EVs [8-10,22]. We conceived the three systemic hUC-MSC infusions as an adjunctive “priming” intervention intended to increase circulating reparative and immunomodulatory mediators and promote resolution of the systemic and local inflammatory response. This systemic strategy was designed to complement the high, spatially targeted dose of EVs administered directly into the rupture gap and tendon stumps. This clinical decision was consistent with preclinical data showing that MSC- and tendon-stem-cell-derived EVs dampen the early inflammatory response after tendon injury, promote M2 macrophage polarization, stimulate tenocyte proliferation and migration, and enhance organized collagen deposition and biomechanical recovery [13], and that hUC-MSC exosomes in particular can direct tendon regeneration via mTOR signaling [16]. Mechanotransductive cues delivered by the progressive rehabilitation program plausibly acted in synergy with the biologic milieu to favor aligned collagen remodeling [22].

From a translational therapeutic standpoint, umbilical cord tissue is an attractive allogeneic MSC source: it is ethically sourced noninvasively from otherwise discarded perinatal tissues, yields cells with high proliferative capacity and low immunogenicity, and lends itself to standardized, scalable manufacturing of both cells and EVs. Most importantly, this cell source has been widely studied and applied in preclinical models demonstrating its safety profile when used both systemically and locally. Realizing this clinical potential nonetheless requires production under good manufacturing practice (GMP) conditions and compliance with the applicable advanced-therapy regulatory frameworks, together with standardized EV characterization and nomenclature in line with the MISEV guidelines of the International Society for Extracellular Vesicles. Furthermore, while this report highlights the potential of cell-free biological therapies, future efforts should prioritize characterizing the molecular payload, such as specific miRNAs or proteins, within these vesicles to better elucidate their role in modulating local macrophage phenotypes and the surrounding inflammatory microenvironment.

Limitations

As an uncontrolled single observation, this report cannot establish causation. Acute complete ATR can heal nonoperatively in selected patients, so spontaneous healing and the concurrent rehabilitation program are unavoidable confounders, and the specific contribution of the systemic cells versus the local EVs cannot be disentangled. Structural assessment relied on qualitative MRI without quantitative tendon-mapping sequences (e.g., T2/T1rho), and the two imaging timepoints preclude characterization of the healing trajectory. Results from a single, healthy, motivated patient may not generalize. These limitations notwithstanding, the case is hypothesis-generating and supports formal evaluation of MSC/EV-augmented nonoperative management for complete ATR. Finally, the biologic product used was not a licensed biologic and was administered outside a registered clinical trial, as individualized clinical care under national regulation; this limits generalizability and precludes any inference about a regulatory-grade benefit-risk profile

Future directions

Prospective controlled studies are needed to define the role, dosing, timing, and delivery route of MSC- and EV-based therapy in ATR, ideally with quantitative MRI (T2/T1rho mapping) and serial ultrasound to track remodeling, standardized EV characterization, and objective isokinetic and return-to-sport metrics. Open questions include the relative contribution of systemic versus local delivery, the optimal biological “window” after injury, and whether the combined approach can safely narrow the re-rupture gap that currently limits nonoperative care.

Patient perspectives

“*When I was told my Achilles tendon was completely torn, I was worried I would need surgery and a long time away from the gym and running. I chose the nonoperative, cell- and vesicle-based treatment and followed the rehabilitation program closely. Recovery was gradual but steady, I never had significant complications, and today I have returned fully to training and jogging without limitations. I am very satisfied with the outcome.*”

## Conclusions

In this single case, a fully nonoperative regimen combining systemic hUC-MSC infusion with ultrasound-guided intralesional hUC-MSC-derived EVs and structured rehabilitation was associated with imaging-confirmed restoration of tendon continuity and complete functional recovery of an acute complete Achilles rupture with a large gap, without complications. While causal attribution is not possible from a single observation, the result supports the biological plausibility of MSC/EV-augmented nonoperative management and justifies controlled prospective investigation. 
